# “Becoming an independent feeder”: infant’s transition in solid food introduction through baby-led weaning

**DOI:** 10.1186/s12919-020-00198-w

**Published:** 2020-12-08

**Authors:** Ayu Fitria Utami, Dessie Wanda, Happy Hayati, Cathrine Fowler

**Affiliations:** 1 Faculty of Nursing, Universitas Indonesia, Depok, Indonesia; 2 Department of Pediatric Nursing, Faculty of Nursing, Universitas Indonesia, Depok, Indonesia; 3 Faculty of Health, University of Technology Sydney, Sydney, Australia

**Keywords:** Baby-led weaning, Complementary feeding, Infants’ responses, Solid foods

## Abstract

**Background:**

Baby-led weaning (BLW), a method for introducing complementary foods, has become popular because it is considered beneficial for infants.

**Methods:**

This study investigated the experiences of mothers when using BLW in Jakarta, Indonesia using a qualitative descriptive approach. Thirteen mothers participated who had introduced complementary feeding using BLW for a minimum of 6 months. Semi-structured interviews and thematic analysis was used to work with the data.

**Results:**

Three themes were identified: avoiding being a ‘picky’ eater; infants gagging and choking; and becoming independent feeders.

**Conclusion:**

Further research related to the growth and development of baby-led weaning infants in Indonesia is recommended.

## Background

Learning to accept and eat complementary (solid) foods is an essential and major milestone for infants. By 6 months of age, infants require an introduction to a variety of foods, as breast milk and infant formula no longer fulfill their nutritional needs [1, 2]. If the introduction of complementary foods is delayed, infants are at increased risk of developing: growth problems such as ‘stuntedness’ or low growth, delayed motor and mental development, neurological and mental fatigue, frequent diarrhea, and a lack of macro and micro nutrients in their body [2, 3].

To support the acceptance of complementary foods at 6 months, the infant’s protective tongue thrust reflex is no longer present and they are learning to chew, are able to sit and have good head control and improved hand eye coordination [4]. As an infant’s awareness increases they will voluntarily place items in their mouths and show an interest in reaching for parents’ food and cutlery at mealtimes [5]. These developmental shifts and a demonstrated interest in food signals the infant’s readiness for being provided with complementary foods.

The introduction of complementary foods triggers a gradual process of weaning the infant from a reliance on breast milk or infant formula to complementary foods. Traditionally, these introductory foods are in a pureed form becoming more varied as the infant learns to accept different textures and flavours [6, 7]. Critically, breastfeeding or infant formula still remains an essential part of the infant’s diet until at least 12 months of age [3].

Parents are regularly provided nutritional, developmental and cultural advice about the timing and method to use, sequence, types and textures of appropriate introductory foods from health professionals, family, friends and other sources. Foods are usually offered in accordance with government guidelines in their country [8, 9]. Meanwhile in Indonesia, guidelines are provided by the Indonesia Pediatric Society about Implementing Complementary Feeding. This guideline refers to complementary feeding principles based on the World Health Organization (WHO) guidelines [10].

Baby-led weaning (BLW) is growing in popularity with parents as it provides an alternative approach of infants being presented with the family diet and being allowed to self-feed rather than the gradual introduction of pureed foods and Traditional Spoon Feeding (TSF) by an adult [11, 12]. This BLW method allows the infant to lead the eating process by encouraging independence in choosing which food and the amount they will eat [5, 12, 13]. BLW presents the foods eaten by the family so that infants experience a family diet [14, 15]. The food offered is manageable to handle and eat. Critically, an adult needs to closely supervise the feeding process and the food offered should minimize the risk of choking, for example avoid crisp hard vegetable and whole nuts.

This article will report a qualitative descriptive study about the experiences of mothers when using BLW. The three themes found within the data to be explored are: avoiding being a ‘picky’ eater; infants gagging and choking; and becoming independent eaters.

## Methods

### Research design

A qualitative descriptive approach was used to explore mothers’ experiences of using BLW to introduce their infants to complementary foods. A qualitative descriptive approach provides “… a comprehensive summary of events in the everyday terms of those events” [16]. It allows researchers to stay close to the data [16]. Ethical approval for this study was gained from the Ethics Committee of the Nursing Faculty at Universitas Indonesia (No. 29/UN2.F12.D/HKP.02.04/2019).

### Participants

In this study, 13 women were recruited. The women had an infant who were at least 12 months old; the oldest child was 5 years, BLW had been used to introduce complementary foods, and the mother is the usual person supervising the infant during mealtimes. The women spoke Bahasa Indonesia and were willing to share their experiences. Prior to agreeing to participate, the women were given an information sheet and an opportunity to think about their willingness to participate. All the women provided verbal and written consent. A snowballing sampling method was used to recruit participants. Thirteen women were recruited through the “BLW Stories”, “Happy Eater”, and “Cacabun” groups on Instagram and WhatsApp platforms, spread throughout the Special Capital Region of Jakarta and surrounding areas. The women were given information sheets and asked to sign a consent form. Recruitment ceased when data saturation was achieved.

### Data collection and analysis

The data were collected using semi-structured interviews conducted in Bahasa Indonesia and took up to one and a half hours in the participants’ houses, shopping centers, cafés, and other places. During the interview the women were asked to reflect on: the reasons for using BLW; the management of BLW; outcomes for the baby; and maternal experience of BLW.

The interviews were digitally recorded and conducted in accordance with interview and confidentially guidelines. All data were deidentified. The verbatim transcripts were returned to the participant to be validation. Each interview was translated into English and back translated to Bahasa Indonesia to ensure translation accuracy.

The theme analysis was guided by the process described by Borbasi and Jackson [17]. Analysis required reading and rereading of the transcripts, using inductive reasoning to identify patterns. The data were independently coded by two researchers to identify patterns and develop categories. These were reviewed to identify similarities and differences; data that were strongly aligned were described and defined as themes [17]. Data collection was stopped once saturation was reached.

## Results

The 13 women interviewed were aged 25-to-36 years old. Eleven women had completed Bachelor degrees and two participants had completed Masters degrees. Ten women worked at home and three women were in the paid workforce as a dentist, advertising assistant and university lecturer. Three main themes were identified within the data during the analysis. Each of these themes consist of several sub-themes as seen in Table 1.

**Table 1:** Themes and Sub-themes

Themes	Sub-themes
1. Avoiding being a ‘picky’ eater	Trying and accepting different foods
	Learning to accept different food textures
2. Infants gagging and choking	Infants gagging and choking
	Gagging as part of the learning process
3. Becoming independent feeders	Learning to be independent
	Focus on eating

### Theme 1: avoiding being a ‘picky’ eater

This theme consisted of two sub-themes: i) trying and accepting different foods; and ii) learning to accept different food textures. This first theme included trying a variety of foods that some infants may not like and reject due to the taste or texture of the food.

#### Trying and accepting different foods

Different maternal attitudes were present to the acceptance or rejection of foods as the infants were learning to eat a range of different foods. Some mothers commenced by labelling their child as a ‘picky’ eater as in the quote from Jane.

There is a stop eating action [when] the child is very ‘picky’ with the foods, if the child says ‘no’. So, what we give … [food] my child eats. If my child doesn’t want to eat … meaning that my child stops eating ...my child is willing to eat later gradually. (Jane)

Jane then provided a contradictory statement about her child’s behaviour saying her child is *very ‘picky’* and refuses the food by saying *no.* Jane goes on to say; *what we give … my child eats* and they are *willing to eat later gradually.* While Jane identifies her child as a ‘picky’ eater she infers later on that with time the infant ate food previously rejected.

Counter to labelling her child as a ‘picky’ eater, Silvana in the following quote rejects this label.

Personally, I don’t label my child as a ‘picky’ eater, but my son, following baby-led weaning, certainly becomes very ‘picky’ or eats [only] certain foods. But for me it is not as a ‘picky’ eater. (Silvana)

Silvana reframes her language from *becomes very ‘picky’* to *eats* [only] *certain foods.* She continues on to refute that her child is a ‘picky’ eater. Contradictions continue during Gabriela’s statement about her son’s eating behaviour.

My son basically doesn’t have problems with his meals, but it will be easy for him to eat without vegetables. (Gabriela)

While Gabriela positions her son as not having a problem with his mealtime eating behaviour she then qualifies this by saying that his preference is not to eat vegetables. The three statements above all provide contradictory elements of the child not wanting to eat certain foods when they are first introduced to them but given time it is recognised that they will eat these foods.

Barbara notes that her son is easy to feed and not ‘*picky’ with his food*.

My son is easy to feed when we take him for a walk outside, and he doesn’t tend to be picky with his foods. (Barbara)

This behaviour is attributed to *when we take him for a walk outside,* there is a possibility that the mother used a form of distraction to encourage her child to eat. While Barbara does not explicitly state that he is ‘picky’ when he is fed inside, her statement could be interpreted as this. In the final quote Margaret firmly states that:

the benefits of this BLW [are] not being ‘picky’, not a ‘picky’ eater

#### Learning to accept different food textures

Learning to eat a variety of foods is an important part of weaning onto a solid food diet. For some infants unfamiliar food textures act as an inhibitor to the acceptance of certain foods.

In the following statement Silvana identifies that:

My son basically doesn’t reject all types of textures. (Silvana)

Silvana’s statement that her son *doesn’t reject all types of textures* implies and confirms that his rejection of some foods maybe linked to different texture types.

While Margaret in the following statement highlights the importance of BLW as preparing the infant to experience and accept a range of food textures.

In my opinion, the benefits of this baby-led weaning … [they] get ready to elevate the texture. (Margaret)

Importantly, Margaret identifies that using BLW helps the infant increases or *elevate* the type of food texture they accept*.*

### Theme 2: infants gagging and choking on food

It was not surprising that the women raised the issues of gagging and choking on food. These two episodes occurring when an infant is feed can be frightening for mothers. This theme consisted of two sub-themes: i) infants gagging and choking; and ii) gagging as part of the learning process.

#### Infants gagging and choking

Gagging is a protective reflex necessary during the early months when learning to eat foods of differing textures. While choking can be both concerning and frightening for mothers.

Choking alhamdulillah [praise be to God] is never experienced by my child. But my son experienced gagging several times, though he was not in extreme trouble. (Laura)

Laura recognises choking as a problem and she is please her child has not had a choking episode. She acknowledges that her son has gagged several time but by the choice of her words and the tone use she is not concerned about these episodes.

Daniela also spoke about gagging as a normal process:

Gagging, [no] gagging never experienced by my child, when I read about gagging, which is normal, just wait for a moment. (Daniela)

Daniela has done some preparative reading about gagging and is prepared with a strategy to manage such an episode.

#### Gagging and choking as part of the learning process

Gagging was identified by some women as part of the learning process needed to learn to eat complementary foods. Daniela has not experienced her infant gagging, though she is not dismissing the possibility of it occurring in the future.

I think it [gagging] doesn’t cause a serious problem for my child, though it can be worse. It is part of the learning process for my child. But it still makes me panic. (Daniela).

Daniela minimizes the impact of gagging as *doesn’t cause a serious problem* and clearly identifies gagging as part of the *learning process for my child*. While for Elaine she has experienced her infant choking.

But alhamdulillah [praise be to God] during complementary feeding, once my child choked because of eating tofu. It perhaps happened because the tofu was cut inside. I am not sure whether he pulled it or something else. (Elaine)

Elaine reflects on the cause of the choking and relates it to the preparation of the tofu and queries her son’s eating behaviour *whether he pulled it.* Though her reflection ends with it could *be something else*.

### Theme 3: becoming independent feeders

All the women identified that their infants were becoming independent feeders as an outcome of BLW. This theme consisted of two sub-themes: i) learning to be independent; and ii) focus on eating.

#### Learning to be independent

The mothers identified that through BLW their infants developed skills that enabled their increasing ability to eat complementary foods.

It is good to stimulate her through finger movements; she can take the food by herself. (Ruth)

Ruth has identified giving her infant the opportunity to pick up food from their feeding tray has assisted in the development of gross and fine motor skills. This enhanced development has been described as facilitating eating independence.

This is expanded on in the next quote, Margaret illustrates the benefits of being provided foods that require an infant to chew food.

This is good for my son since he can chew the foods. He can directly chew foods with rough textures, I mean, such as rice, which is more solid. (Margaret)

Margaret continues to highlight the positive outcomes of BLW of being able to chew foods that are *rough textures* and *more solid.* While another mother directly attributes her infant’s increasing willingness to trying new foods and her developing mealtime independence.

Her willingness, who was in the oral phase, was facilitated. (Anna)

Anna also mentioned *the oral phase*, a phase of development where the infant explores their environment by placing things in their mouth is identified as contributing to the infant’s success in trying new food.

The BLW was identified as increasing their independent behaviour when they had experienced the BLW method.

What I experience … [he] had been able to drink by himself. (Nora)

She can eat with or without [a] spoon. (Ruth)

These two mothers have attributed their infants’ new skills of *drink by himself* and *eat with or without* [a] *spoon.* They identify these skills as important development milestones.

The women spoke of several BLW positive outcomes. These outcomes included independence, involvement and socialization.

I see my child becomes more independent quickly … because my child can eat by herself, so [she] just eats by herself. I like her because she becomes active [in feeding herself]. (Thalia)

While for Thalia her daughter’s increasing eating independence is valued. The reason being given is her daughter’s active and independent involvement in the feeding process.

In the next quote, Daniela was now allowing her son to be involved in meal preparation a further step towards independence and the reduction in parental control.

He also is involved in preparing the meals and ... can choose his own meals. (Daniela)

Of note in Daniela’s statement is that her son choose his own meals. From the tone of her voice this is identified as a positive outcome of BLW. As BLW has a focus on infants being offered a family diet rather than being provided with special foods the need for separate preparation and thought being given to the provision of an alternative infant diet is eliminated.

It also affects me; cooking preparation becomes simpler [laughing]. Then after that we can eat together. (Daniela)

This focus on family meals provides a motivation to eat together as a family. This approach exposes the infant to the social and learning aspects of eating with the family.

Expressing *what he wants* and being *responsible* are identified as key and valued features by Gabriela of her son’s mealtime behaviour.

Little by little he is able to express what he wants. He wants something. He becomes consistent on it. He is responsible … (Gabriela)

These positive outcomes may not all be due to BLW, regardless the women in this study have attributed them to the use of BLW to introduce their infants to complementary foods and the outcome of developing independence with the eating process.

#### Focus on eating

The women valued BLW as it provided a focus on eating rather than the need to distract the child to get them to eat.

When [it’s] mealtime, we directly eat, sit and eat, meaning eating without playing or eating while not doing something else. (Daniela)

Daniela places emphasis on *eat, sit and eat.* The impression is given of her infant being socialized into acceptable mealtime behaviour.

In this quote Ruth directly attributes BLW for providing a trigger for her child to be disciplined.

That’s why [the] BLW method, instead of training children on eating skills, it also triggers my child to be disciplined—eating should be [done while] sitting. (Ruth)

Being disciplined is spoken of as a different approach than *training children on eating skills.* Ruth’s statement provides the impression that she values the ability of her child *to be disciplined* which is equated with sitting while eating.

Being responsive to a child’s hunger or satiation cues or comments is critical as allowing the infant to be in control of their eating behaviour is central to BLW. For Barbara she illustrates how her infant is able to say when he is full.

Now he can be like that; if, for instance, he is already full, he will say that to me: “Mom, I am full!” [imitating her son], then [he is] reluctant to eat any more. (Barbara)

From the statement by the mother she is respectful of his decision that he is no longer hungry.

## Discussion

This research study has explored the experiences of Indonesian mothers when using BLW to introduce their infants to complementary foods. BLW has been accepted by the mothers as having several positive outcomes for their child’s developing mealtime independence and being less likely to be ‘picky’. The mothers identify there were gagging and choking risks related to BLW. However several mothers felt being educated about the management of choking and gagging which helped them manage these situations.

The portrayal of a child as a ‘picky’ eater is when infants refuse certain foods or food groups [18]. The terminology ‘picky’ eaters has also been used to describe fussy eating [19]. This problem is characterized by infants’ reluctance to try foods that can be unfamiliar. Parents often misinterpret their infants’ cue and continue to encourage them to eat rather than understanding that it might be a signal of satiation from the child [20]. On the other hand, when parents apply responsive feeding techniques their infants tend to enjoy food more and have a longer tolerance for waiting to eat a meal. When infants enjoy their food they are likely to accept various types of food [20].

The women in this study observed that their infants were not ‘picky’ eaters as a result of using BLW. While a couple of the women started to talk about the child being ‘picky’ but then dismissed this view. This observation is consistent with other research that purported BLW to prevent infants from becoming ‘picky’ eaters as they are exposed to family meals and eat at the same time as other family members increasing the social impact of eating [5, 13, 15]. The women spoke in positive terms about their infants’ willingness to: try and accept a variety of foods including vegetables; accept different food textures, and easily accommodate eating when not at home. Using BLW with their infants showed an understanding that is in line with the principle of responsive feeding. While some of the women constructed their child’s leaving food or eating slowly as a matter of a child’s eating process rather than being ‘picky’. Concerns were not raised that their children were not eating an adequate diet.

Since BLW became popular, it has been raised that BLW could compromise the infants health due to: inadequate amounts of iron rich foods being eaten; increased anemia risk; the potential for choking; and increased mess [12]. Parents need to be aware of these nutritional concern and ensure the food types offered optimize their nutritional intake. When infants reach 12 months of age they should be eating the same food as the family [21]. The effects of the early introduction of vegetables makes it easier to accept and consume vegetables early in life and throughout childhood [22].

In this study, nine women said their infants had experienced gagging while two had experienced their infant choking. While choking increases the risk to the child if not immediately attended too. The women in this study had already learnt about the difference between gagging and choking and how to manage the situation; resulting in identifying gagging as a normal process. Importantly, both BLW [23] and TSF infants can be exposed to choking risks when give certain foods; for instance hard textured foods [8]. If choking occurred the women had anticipated intervening by patting their infants’ back and trying to calm them down. Critically, limiting the type of food to avoid choking still poses a risk if parents do not pay close attention to their child at mealtimes. BLW has been demonstrated to be safe as TSF [24, 25]. Regardless of the method used to introduce complementary feeding, women must be aware of information about safe foods for infants and how to handle choking episodes [24].

The infants in this study were identified as being more independent at family mealtimes and enjoying the eating process as they: controlled their own eating; know when they are hungry and full; did not hold food in the mouth; and were focused on the task of eating. The women provided various forms and textures of food enabling their infants to explore their foods. The infants were described as happier and more enthusiastic when mealtime arrived. These outcomes were in-line with the study of Komninoue et al. as the parents who used BLW were more likely to sit with their infants and share the same food during the eating process. The parents became their infants’ “eating friends” [26]. Women who used BLW are identified as having multiple positive outcomes such as teaching infants to eat independently and also providing space for infants to learn to regulate their eating process as an important and ongoing skill for healthy eating [27].

## Conclusions

Infants who are weaned using BLW show certain responses of increased independence and eating without fuss. The infants eat with the family and share the same foods. The infants’ increasing independence in this study arose due to the opportunities they were provided to regulate the amount of food eaten and the overall feeding process. The women encouraged their infants to feed themselves instead of being fed by others. The infants became confident and eventually independent eaters.

Infants at times were likely to experience gagging and choking. When gagging occurred the women motivate their infants to be able to independently overcome gagging, and as a result they successfully manage the gagging episode. These responses had been enhanced due to the women learning how to properly handle choking incidents.

## Data Availability

Interview transcripts are available from the corresponding author upon reasonable request.

## Abbreviations

BLW: Baby-Led Weaning
TSF: Traditional Spoon Feeding
WHO: World Health Organization
